# New Formula for the Hydrogen-Bonding Hansen Component of Methanol, Ethanol, and *n*-Propanol for Non-ambient Conditions—Application in Gas Antisolvent Fractionation-Based Optical Resolution

**DOI:** 10.1021/acsomega.1c02223

**Published:** 2021-07-12

**Authors:** Máté Mihalovits, Márton Kőrösi, Edit Székely

**Affiliations:** Department of Chemical and Environmental Process Engineering, Budapest University of Technology and Economics, Budapest 1111, Hungary

## Abstract

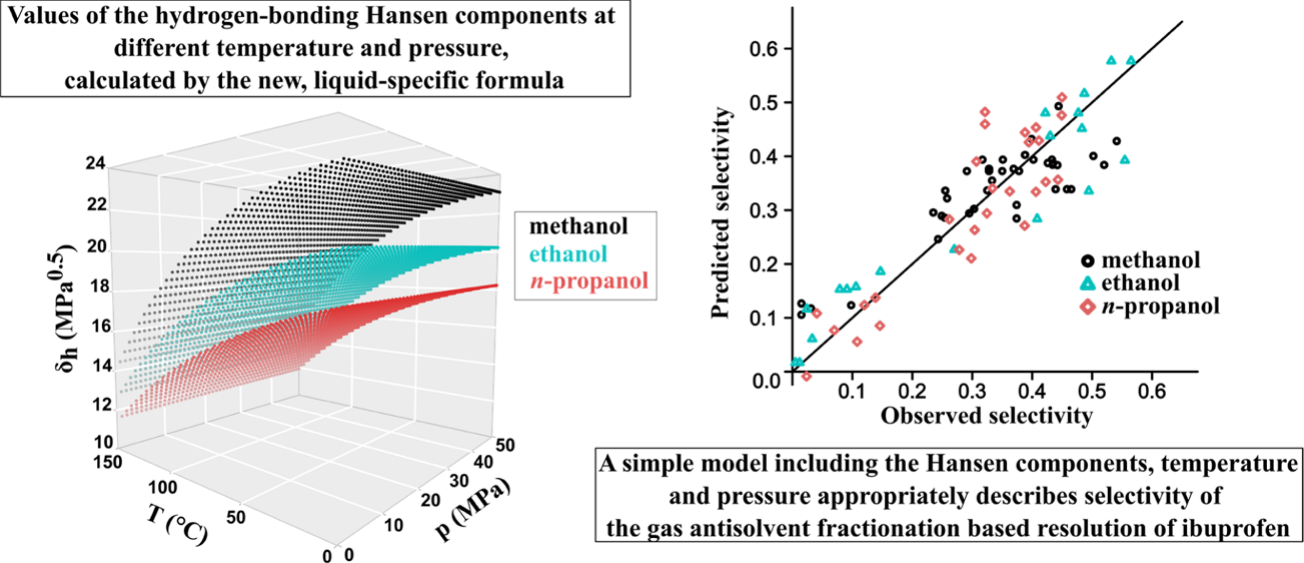

Optical resolution by diastereomeric salt formation based on gas antisolvent fractionation is influenced by the chemical equilibrium of the salt formation, the solubility, and the extraction of the compounds. Selectivity, also known as resolution efficiency, is highly solvent-dependent and is also affected by process parameters both in atmospheric and supercritical processes. For the first time in the literature, a mathematical model that employs all three Hansen parameters and operating parameters is constructed to describe the selectivity of a gas antisolvent fractionation process. The satisfying goodness of fit of the models suggests that the outcome of the three subprocesses in the gas antisolvent fractionation process (i.e., salt formation reaction, precipitation, and extraction) can be described in a single model. A new formula for pressure and temperature correction of the hydrogen-bonding component of the Hansen parameter for non-ambient conditions for liquid methanol, ethanol, and *n*-propanol is also suggested in this paper.

## 1 Introduction

Solubility parameters are physical–chemical indexes that characterize the intermolecular forces in substances. Application of solubility parameters enables to predict to what extent two substances can be mixed. They are widely used in different fields where solubility is of importance, for example, cocrystal formulation (Mohammad et al.^1^), polymer chemistry (Hansen^2^), the solubility of bitumen (Redelius^3^), extraction (Srinivas et al.^4^), and coating formulation (Hansen^5^). The concept of solubility parameters was first discussed by Hildebrand and Scott.^6^ The Hildebrand solubility parameter, δ (MPa^0.5^), is defined as
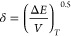
1where Δ*E* is the molar energy of vaporization (J/mol), *V* is the molar volume (cm^3^/mol), and (Δ*E*/*V*)_*T*_^0.5^ is the square root of the cohesive energy density. Although the Hildebrand parameter is a widely applied and convenient index for dissolution in liquids with nonpolar and slightly polar nature (e.g., solubility of polymers in organic solvents), the application of the parameter is limited due to the lack of capability to differentiate between the types of intermolecular interactions. Hansen^7^ extended the concept of the Hildebrand solubility parameter by partitioning the molar energy of vaporization into three components
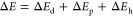
2where the subscripts indicate the type of the intermolecular interaction with which the component contributes to the total molar energy of vaporization: d stands for the dispersion, p stands for the polar, and h stands for the hydrogen-bonding interaction. The Hansen components (i.e., Hansen parameters) are defined as the square root of the ratio of the contribution of the corresponding component of Δ*E* and the molar volume (*V*), that is
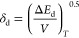
3
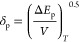
4
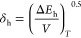
5

The Hansen solubility parameter is defined as
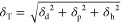
6where δ_T_ is the total Hansen parameter (MPa^0.5^), δ_d_^2^ (MPa) is the contribution of the dispersion forces, δ_p_^2^ (MPa) is the contribution of the dipole forces, and δ_h_^2^ (MPa) is the contribution of the hydrogen-bonding forces to the total Hansen parameter.

The closer the values of the solubility parameters of different substances, the better the dissolution of the components in each other. Hansen^8^ developed the equation for “distance” between Hansen parameters of two components
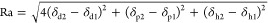
7where the values with the subscript of 1 correspond to one of the components in the mixture and the values with the subscript of 2 correspond to the other component in the mixture. Smaller Ra indicates substances that dissolve each other better, compared to those with higher Ra. Substances with the same solubility in a given solvent can be found on an ellipsoid around the Hansen parameters of the solvent, in a three-dimensional plot of the Hansen components. When the scale of the axis of δ_d_ is half of the scales of the other two axes, the ellipsoid becomes a sphere which is referred to as Hansen solubility sphere.

Hansen^9^ discussed the determination of the Hansen parameters for ambient conditions by solubility experiments, theoretical calculations, and different group contribution methods. The group contribution methods provide a convenient estimation for the Hansen parameters as no experimental results or theoretical considerations are required. The most applied methods are those developed by Stefanis and Panayiotou,^10,11^ Hoftyzer and van Krevelen,^12^ and Hoy.^13^ In these methods, the Hansen components are estimated based on the structural groups present in the molecule. The methods were shown to have good descriptive accuracy and may be applied when no experimental determination is available for the investigated substance.

Solubility parameters may also be applied in processes involving supercritical solvents. Supercritical carbon dioxide (scCO_2_) has already been applied in the industry as an extraction solvent, a solvent in dyeing and impregnation processes and as a reaction medium. Besides its non-flammability and non-toxicity, another favorable property of scCO_2_ is that its physical–chemical parameters (such as density and viscosity) depend on pressure and temperature. This adjustability can, for example, be exploited in fractioned separation after supercritical fluid extraction, where different families of components may be precipitated in separators operating at different pressures. Several other application possibilities have also been developed (from polymer foaming through micronization to the application of carbon dioxide as a coolant). A review of the application of scCO_2_ in the industry is available by Knez et al.^14^

The effects of the solubility parameters involving supercritical solvents (e.g., scCO_2_) are discussed, for example, by Lagalante et al.,^15^ Wai et al.,^16^ Tirado et al.,^17^ Sánchez-Camargo et al.,^18^ Tirado and Calvo,^19^ Seo et al.,^20^ and Pereira et al.^21^ It was found in these studies that the solubility parameters correlate with the measured response of the processes; however, they are difficult to be distinguished from the effects of pressure and temperature. In these investigations, simple correlation studies between the measured attribute and the solubility parameters are considered, and more complex mathematical tools and approaches are hardly applied. Moreover, no investigation in the literature aims to describe the process with a mathematical model using the Hansen components or the total Hansen parameter. To the best knowledge of the authors, there is only one exception which is actually a previous study of the authors.^22^ In that study, model building was applied using the total Hansen solubility parameter, temperature, and pressure to describe the selectivity of the gas antisolvent fractionation (GASF)-based optical resolution of ibuprofen with a mixture of scCO_2_–methanol and scCO_2_–ethanol. The GASF aims to selectively precipitate one or more components from their organic solution by the addition of the pressurized carbon dioxide while keeping the other component(s) dissolved and extractable (Kordikowski et al.,^23^ Martín and Cocero,^24^ and Székely et al.^25^). In the GASF-based optical resolution, the main goal is to separate the enantiomers of a racemate with the highest purity (i.e., enantiomeric excess) and yield at the same time. This method is discussed in more detail, for example, in the work of Kőrösi et al.^26^

To advance the description and prediction of gas antisolvent-based optical resolution, in this paper, the possibility of the construction of a mathematical model using the Hansen components and the operating parameters is investigated. A simple model may help decrease the number of experiments needed when a new GASF system is being investigated as the potential sources of effects might be general for different GASF systems. Also, it may help investigate new gas antisolvent processes more effectively and find optimum settings for a process in energy-, time- and resource-efficient way. Compared to the previous work, additional experimental results from GASF-based resolution of ibuprofen with the scCO_2_–*n*-propanol mixture and GASF-based resolution of mandelic acid with the scCO_2_–methanol mixture (Kőrösi et al.^26^) are used in the evaluation in this paper as well.

The Hansen components when applied for non-ambient conditions are to be corrected by temperature and pressure. The formulas for the corrections developed by Williams et al.^27^ are widely applied in the literature; however, the accuracy of the formula of correction of the hydrogen-bonding component (δ_h_) is questionable. New formula for correction of δ_h_ is suggested in this paper for the organic solvents applied in the experiments considered in this paper: methanol, ethanol, and *n*-propanol.

## 2 Materials and Methods

### 2.1 Materials and Experimental Methods

The materials and the experimental methods are not discussed here in detail due to being available in previous studies of the research group of the authors of this paper (Kőrösi et al.^26^ and Lőrincz et al.^28^). The summary of the four series of experiments considered in this paper can be found in Table 1.

**Table 1:** Summary of the Considered Experiments

organic solvent	racemate	resolving agent	number of experiments	temperature (°C)	pressure (MPa)
methanol	mandelic acid	(*R*)-1-methylbenzylamine	20	35–55	12–20
methanol	ibuprofen	(*R*)-1-methylbenzylamine	40	35–55	10–21
ethanol	ibuprofen	(*R*)-1-methylbenzylamine	19	35–45	10–20
*n*-propanol	ibuprofen	(*R*)-1-methylbenzylamine	27	35–45	10–20

The materials, the applied method, and the results for the GASF-based resolution of mandelic acid with (*R*)-1-methylbenzylamine can be found in the study by Kőrösi et al.,^26^ while the experiments with ibuprofen and methanol can be found in the study by Lőrincz et al.^28^ Although the materials and the applied method of the experiments of ibuprofen with ethanol and propanol are not published, those are the same as in the study by Lőrincz et al.^28^ Additionally, ethanol and *n*-propanol (99.98%) were purchased from Molar Ltd. The results of the experiments with ibuprofen, ethanol, and *n*-propanol are not published, and some of the data from the experiment with ibuprofen and methanol are unpublished as well. The data for every experiment are provided in the Supporting Information tables.

### 2.2 Calculation of the Selectivity

In the GASF experiments, involving the precipitation of diastereomeric salts, considered in this paper the main goal was to separate the enantiomers of a racemate with the highest purity (i.e., enantiomeric excess) and yield at the same time. Selectivity (or resolvability), as suggested by Fogassy et al.,^29^ was calculated as the product of the enantiomeric excess and the yield. All of the experiments were conducted with a molar ratio of 0.5 between the resolving agent and the racemate. The yields (both of the raffinate, i.e., the crystalline product and the extract) were referred to as a theoretical maximal mass, assuming complete insolubility of the salts and complete extraction of the unreacted enantiomers. The formulas for calculating selectivity are the following
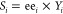
8
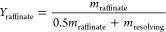
9
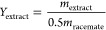
10
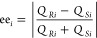
11

In the above formulas, *S* stands for selectivity, while the subscript *i* indicates that the formula is the same for the raffinate and the extract. The yield, marked by *Y*, is defined in different ways for the raffinate and the extract. In the formulas, *m* denotes the mass (g) of the product or starting material specified by the subscript words. The enantiomeric excess (ee_*i*_) of the acids (ibuprofen or mandelic acid) in the products was determined based on capillary electrophoretic measurements. In the formula, *Q* stands for the quantity (e.g., mass, concentration, and chromatographic peak area) of the enantiomers [(*R*) and (*S*)] specified in the subscripts. The formula was used identically in the case of the solid raffinates and the extracts of the GASF processes. The diastereomeric salts forming in the reaction of the acids and the enantiomerically pure (*R*)-1-methylbenzylamine decomposed under the circumstances of the analyses. Hence, ee is suitable to describe the average enantiomeric excess of the acid found in the solid product.

### 2.3 Temperature and Pressure Correction of the Hansen Parameters

The solubility parameters were originally developed for ambient temperature and pressure. For the extension of the application of the parameters for non-ambient conditions, different formulas were suggested. Marcus^30,31^ derived the formula for supercritical water and supercritical methanol, while the most widely applied temperature and pressure correction (including the supercritical region) of the Hansen parameters is derived by Williams et al.,^27^ based on the studies of Hildebrand and Scott^1^ and Hansen and Beerbower.^32^ It should be noted that Williams et al.^27^ discussed the formulas using scCO_2_ as an example, and the generalization of the formulas is questionable. Nonetheless, these formulas are applied widely in the literature regardless of the substance for which the correction is used.

According to Williams et al.,^27^ the parameters for non-ambient conditions are calculated as follows
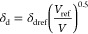
12
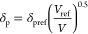
13
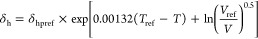
14where δ_dref_, δ_pref_, and δ_href_ are the reference Hansen parameters (MPa^0.5^) and *V*_ref_ (cm^3^/mol) is the molar volume at the reference temperature *T*_ref_ (°C) and reference pressure *p*_ref_ (MPa). Reference values for numerous liquids can be found in the work of Hansen.^9^ In most cases, these values are given for *T*_ref_ = 25 °C and *p*_ref_ = 0.1 MPa. An exception is the reference values of CO_2_ which are given for *T*_ref_ = 25 °C and *V*_ref_ = 39.13 cm^3^/mol and thus *p*_ref_ = 91.7 MPa. It can be seen from the formulas that increasing pressure (through the molar volumes) and decreasing temperature result in increased values of the Hansen components.

The derivation of the formula of δ_p_ does not employ any assumption regarding the liquid, therefore can be used universally, while the formula of δ_d_ was derived for normal (nonassociating or van der Waals) liquids. It is not evident that the formula for δ_h_ is universal and thus should be applied with caution. In the derivation of the formula for δ_h_, the hydrogen bonding contribution to the total cohesive energy is applied. This contribution may be estimated by measuring the difference in the heat of vaporization of the investigated liquid (which shows strong hydrogen bonding, e.g., alcohols) and that of the hydrocarbon (or other nonpolar) homomorph at the same reduced temperature. In this approach, as discussed by Bondi and Simkin,^33^ the dipole energy contribution is absorbed into the hydrogen-bond energy contribution, while the dispersion energy contribution of the −CH_3_ and the −OH groups are taken to be equal. To justify this approach, Bondi and Simkin referred to different exploratory calculations, for example, Pauling.^34^ Pauling^34^ discussed the hydrogen-bond strength for water and different alcohols and stated that one-fifth of the energy contributions can be attributed to the van der Waals forces, and the rest represents the hydrogen-bond strength. Also, it was discussed that based on enthalpies of sublimation and formation of dimers, trimers, and tetramers, the hydrogen-bond energy contribution is in the range of 5000–6000 cal/mol in the case of water and aliphatic alcohols which agrees with the values given in the erratum of Bondi^35^ (Table 5).

The values, as listed in Table 2 (from Bondi and Simkin^33^), were used by Williams et al.^27^ in his derivation of the formula for δ_h_. The hydrogen-bonding parameter, *E*_h_, is actually the hydrogen bond increment in the molar heat of vaporization caused by the given functional group. The constant in the exponent of eq 14 is to be obtained by the following formula
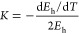
15

**Table 2:** Hydrogen-Bonding Parameter and Its Temperature Derivative

functional group	*E*_h_ (cal/mol), hydrogen-bonding parameter	dEh/dT (cal/mol °C)
–OH (aliphatic)	4650 ± 400	–10
–NH_2_ (aliphatic)	1350 ± 200	–4.5
–CN (aliphatic)	550 ± 200	–7.0
–COOH (aliphatic)	2750 ± 250	–2.9

In Williams’ calculation, *K* is 0.00132 and obtained by taking the average of d*E*_h_/d*T* values and the average of the hydrogen-bonding parameters over the different functional groups, as listed in Table 2, and substituting them in the numerator and denominator of eq 15, respectively.

Therefore, Williams’ formula for the temperature and pressure correction of δ_h_ applies a generalization in which the hydrogen-bonding strength is taken as the average of the hydrogen-bonding strength of the investigated functional groups, as listed in Table 2, independently of the functional groups actually present in the substance for which the formula is used.

#### 2.3.1 Correction for Supercritical CO_2_

The formulas in eqs 12 and 13 can be applied for the temperature and pressure correction of δ_d_ and δ_p_ for scCO_2_. The reported reference values of CO_2_ are δ_dref_ = 15.6 MPa^0.5^, δ_pref_ = 5.2 MPa^0.5^, and δ_href_ = 5.8 MPa^0.5^. In the formula of δ_h_, the application of the mean heat of vaporization for the investigated functional groups, as listed in Table 2 lacks theoretical justification. Nevertheless, there is no available information regarding the *E*_h_ and d*E*_h_/d*T* for scCO_2_, and Williams’ formula is widely applied in the literature, and thus, the practice of the application of this formula is followed in this paper as well. The temperature and pressure corrected Hansen parameters based on eqs 12–14, and with the substitution of *V*_ref_ with 39.13 cm^3^/mol and *T*_ref_ with 25 °C, are then
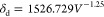
16
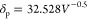
17
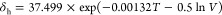
18where *V* (cm^3^/mol) is the molar volume of CO_2_ for the desired temperature and pressure and *T* (°C) is the desired temperature. The values of molar volume for scCO_2_ are obtained from NIST Chemistry Webbook.^36^

#### 2.3.2 Correction for Liquid Methanol, Ethanol, and *n*-Propanol

The formulas in eqs 12 and 13 can be applied for the temperature and pressure correction of δ_d_ and δ_p_, respectively, for liquid organic solvents as well. The reference Hansen parameters of the organic solvents at *T*_ref_ = 25 °C are obtained from Hansen^9^ and can be found in Table 3. The calculation of reference molar volumes is discussed in Section 2.3.3.

**Table 3:** Reference Hansen Parameters at *T*_ref_ = 25 °C and *p*_ref_ = 0.1 MPa

	δ_dref_ (MPa^0.5^)	δ_pref_ (MPa^0.5^)	δ_href_ (MPa^0.5^)
methanol	15.1	12.3	22.3
ethanol	15.8	8.8	19.4
*n*-propanol	16.0	6.8	17.4

Modification of eq 14 is used in this paper to obtain solvent-specific temperature and pressure correction of δ_h_ for liquid methanol, ethanol, and *n*-propanol. The derivation of the modified formula can be found in Section 3.1. Here, only the derived formulas are shown
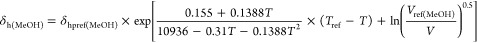
19
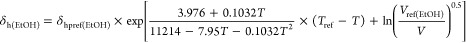
20
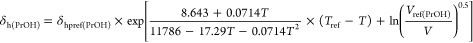
21

The δ_hpref_ values can be found in Table 3, *T*_ref_ = 25 °C, while *T* (°C) is the desired temperature for which the parameter is to be calculated and *V* (cm^3^/mol) is the molar volume at the desired temperature and pressure.

#### 2.3.3 Temperature and Pressure Correction for Molar Volume, *V*

Molar volume is required to calculate the Hansen parameters at different temperatures and pressures. The Tait equation may be applied to estimate density (ρ) and thus molar volume (*V*) for liquids. The Tait equation is the following
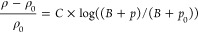
22where ρ (g/cm^3^) and ρ_0_ (g/cm^3^) are the densities of the liquid at the required temperature and required pressure *p* (MPa) and at the required temperature and pressure *p*_0_ (MPa), respectively. *B* and *C* are parameters, and *B* is the temperature-dependent parameter. *p*_0_ is usually taken to be 0.101 MPa, and thus, ρ_0_ is the atmospheric-pressure density, at the given temperature. Different formulas for the calculation of the parameters *B* and *C* can be found in the literature from which two are discussed here. Assael et al.^37^ gave a method to estimate *B* and *C* to use in the Tait equation to calculate the density of liquid *n*-alcohols, from methanol to *n*-decanol, in the pressure range of 0.1–100 MPa. For the construction of the model, the data set is obtained by collecting data from different sources in the literature. Cano-Gómez et al.^38^ used the same data set and gave a method to estimate *B* and *C* which can be applied more generally for *n*-alcohols and at higher pressure as well. The model of Cano-Gómez et al.^38^ is more accurate on average in fitting to the experimental results. However, the authors of this paper found that the model of Assael et al.^37^ gives more accurate results for the range of pressure relevant in this paper (10–20 MPa), and thus, the formula of Assael et al.^37^ is applied here. It should be noted that both models have ±3 kg/m^3^ (±0.4%) error at max in the range of 10–20 MPa when compared to experimental values, and thus, both are accurate estimations.

According to Assael et al.^36^ in eq 22, *C* is a constant with the value of 0.2, and *B* is computed as
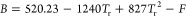
23where *T*_r_ is the reduced temperature and *F* is 11.8 for methanol and 0.015*n*(1 + 11.5*n*) for ethanol to *n*-decanol, where *n* is the number of carbon atoms in the alcohol.

The density at atmospheric pressure, ρ_0_, in eq 22 can be obtained for different temperatures by the formula derived by Cibulka^39^
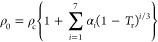
24where ρ_c_ is the critical density and α_*i*_ is a variable. The formula is applicable only in the temperature range of around 170–500 K, which is below the supercritical region of the considered alcohols. Accordingly, this estimation method is appropriate only for liquid phase alcohols. It should be noted that Assael et al.^37^ mispresent this formula in their paper, which leads to great differences in the calculated densities. The values of α_*i*_, critical temperature, and density for methanol, ethanol, and *n*-propanol can be found in Table 4 (based on Cibulka^39^ and Assael et al.^37^).

**Table 4:** Parameters for the Estimation of the Density at Atmospheric Pressure

	*T*_c_ (K)	ρ_c_ (g/cm^3^)	α_1_	α_2_	α_3_	α_4_	α_5_	α_6_	α_7_
MeOH	512.6	0.272	2.6278	–4.0474	15.8343	–22.5066	10.9160	0.3048	0
EtOH	513.88	0.276	–0.9926	38.0287	–181.1172	445.9045	–588.5184	393.9196	–104.3400
PrOH	536.74	0.274	0.9405	12.9442	–53.9519	113.6388	–113.8656	43.3832	0

ρ_0_ is estimated by eq 24 for a given temperature and afterward, ρ can be calculated for that temperature and the desired pressure using eq 22. From that, the molar volume is obtained as *V* = *M*/ρ, and the reference molar volume is obtained as *V*_ref_ = *M*/ρ_0(*T*=298.17K)_ where *M* (g/mol) is the molar mass of the given alcohol.

#### 2.2.4 Calculation of the Hansen Parameters for Mixtures

Each of the Hansen components of the mixture of scCO_2_ and the co-solvent is calculated as (Barton^40^)
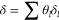
25where δ is the considered Hansen parameter of the mixture, δ_*l*_ is the considered Hansen parameter of the *l*th component in the mixture, and θ_*l*_ is the volume fraction of the *l*th component. According to Hansen,^9^ it may be assumed that there is no volume change upon mixing the solvents when no specific data are available, and thus, this approach may be used. The volume fractions when there are two components in the mixture are obtained as
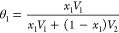
26and
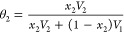
27where *x*_1_ and *x*_2_ are the mole fractions of the first and second component, respectively, and *V*_1_ and *V*_2_ are the molar volume of the first and second components respectively.

To calculate the Hansen components of methanol, ethanol, and *n*-propanol for non-ambient conditions, the points listed here are to be followed:

(1) Calculate the atmospheric density of the liquid using eq 24
(2) Calculate *B* according to eq 23
(3) Calculate the density of the liquid for the desired temperature and pressure using the Tait equation (eq 22)
(4) Calculate the molar volume using the obtained density and *V* = *M*/ρ and the reference molar volume using the density at 0.101 MPa and 25 °C and *V*_ref_ = *M*/ρ_0(*T*=298.15K)_
(5) Calculate the Hansen components by eqs 12, 13, and 19–21

## 3 Results and Discussion

### 3.1 Derivation of the New Formula for the Temperature and Pressure Correction of δ_h_

Similar to Williams’ approach, the results of Bondi and Simkin^33^ are considered here. Bondi and Simkin used the experimental data from Rossini^41^ and Fiock^42^ to obtain *E*_h_ parameters for different alcohols (including methanol, ethanol, and *n*-propanol) at different temperatures. The corrected data for methanol, ethanol, and *n*-propanol can be found in the erratum of Bondi and Simkin^35^ and are listed here in Table 5.

**Table 5:** *E*_h_ (cal/mol) Values from the Erratum of Bondi and Simkin at Different Temperatures

	*T* (°C)					
alcohol	0	30	60	90	120	150
methanol	5500	5350	5200	4900	4500	3850
ethanol	5600	5450	5200	4800	4400	3850
*n*-propanol	5900	5600	5200	4900	4300	3800

The hydrogen-bonding parameter for the −OH group, as listed in Table 2, is the average of the hydrogen-bonding parameters of 10 different alcohols (from which only methanol, ethanol, and *n*-propanol are considered here) at an arbitrary temperature of 100 °C. d*E*_h_/d*T*, as listed in Table 2, is the slope of the common regression line, fitted on the *E*_h_ values of the different alcohols at different temperatures. In the studies of Bondi and Simkin,^33,35^ the data are available for each alcohol and for a range of temperature, and thus, liquid-specific *E*_h_ and d*E*_h_/d*T* values can be obtained for any temperature within the range.

Four modifications are applied in this paper for Williams’ formula to obtain *E*_h_ and d*E*_h_/d*T* specifically for methanol, ethanol, and *n*-propanol at different temperatures. First, only the hydrogen-bonding parameter for the −OH group is considered as the other functional groups are not relevant for aliphatic alcohols when Hansen parameters are considered. Second, a regression curve is fitted on values, as listed in Table 5, for each alcohol instead of the common line to enable the calculation of d*E*_h_/d*T* separately for each alcohol. Third, based on the visualization of the observation, as listed in Table 5, a linear model may not be appropriate to describe the relationship between temperature and *E*_h_, and due to the small sample sizes, it is hard to test the appropriateness of the linear fits. However, when a linear model is fitted in each sample, the mid-range observations tend to be above the estimated line, while the earliest and latest points tend to be below the estimated line. This suggests the need for a quadratic term, and thus, a quadratic model is used instead of the linear one. Finally, the temperature effect on *E*_h_ and d*E*_h_/d*T* is taken into account instead of using only the values estimated at 100 °C.

Based on the data, as listed in Table 5, the quadratic models describing the effect of temperature on *E*_h_ for the three alcohols considered in this paper are
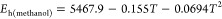
28
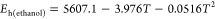
29
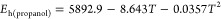
30where *T* is the temperature (°C). From that, the temperature derivates are
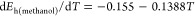
31
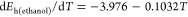
32
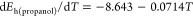
33

In Table 6, the values of *E*_h_ and d*E*_h_/d*T* are given using the above equations for methanol, ethanol, and *n*-propanol for the temperature range relevant for the GASF experiments in this paper. While the values of *E*_h_ are rather close to each other at a given temperature, the differences in the derivatives are not negligible.

**Table 6:** Estimated Values of *E*_h_ (cal/mol) and d*E*_h_/d*T* (cal/mol °C)

		*T* (°C)				
alcohol		35	40	45	50	55
methanol	*E*_h_	5378	5351	5321	5288	5251
	d*E*_h_/d*T*	–4.99	–5.68	–6.37	–7.06	–7.75
ethanol	*E*_h_	5405	5366	5324	5279	5232
	d*E*_h_/d*T*	–7.59	–8.10	–8.62	–9.14	–9.65
*n*-propanol	*E*_h_	5547	5490	5432	5372	5310
	d*E*_h_/d*T*	–11.14	–11.50	–11.86	–12.21	–12.57

**Figure 1:**
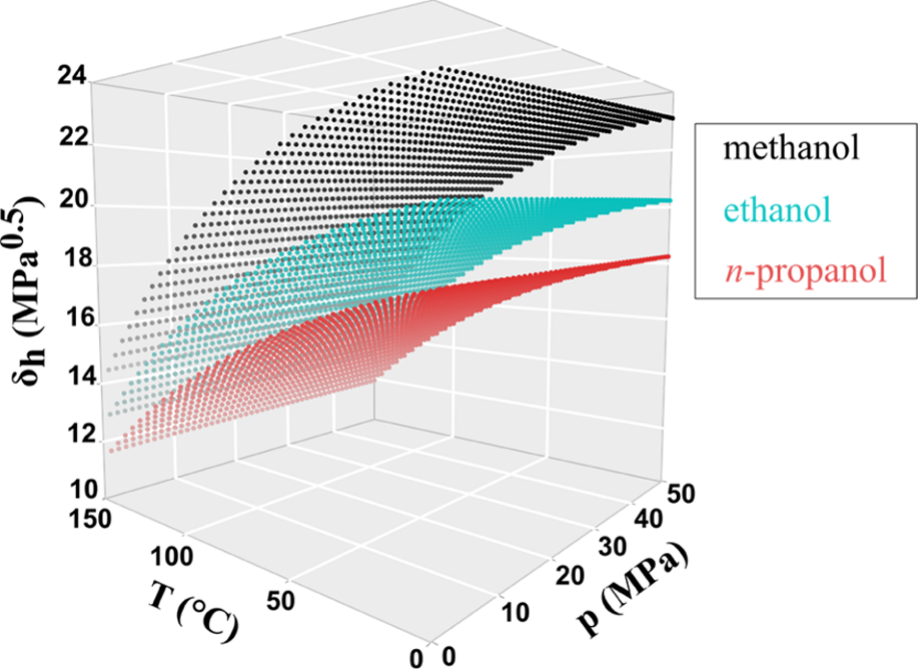
Calculated hydrogen-bonding components at different pressures and temperatures.

**Figure 2:**
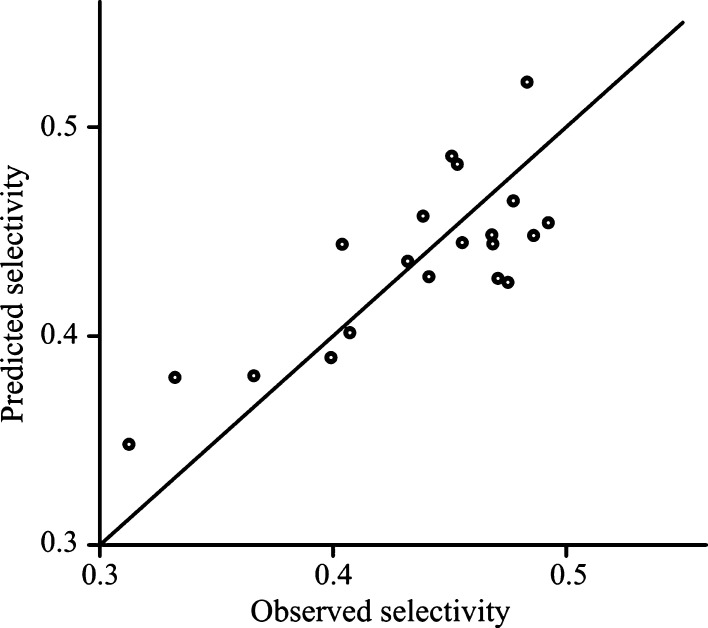
Goodness-of-fit using the OLS model for experiments with mandelic acid.

Recall the formula derived by Williams
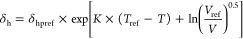
34where *K* is obtained by eq 15. In Williams’ approach, *K* is universal and equals to 0.00132, while the derived formulas in this paper (eqs 19–21) are obtained by calculating *K* with the liquid specific values of *E*_h(alcohol)_ and d*E*_h(alcohol)_/d*T* from eqs 28–30 and 31–33, respectively. It should be noted that *E*_h_ and d*E*_h_/d*T* are estimated based on observations at the temperature range of 0–150 °C. While estimation by interpolation is justified, the derived formulas might be questionable above 150 °C, and extrapolation should be applied with caution. The calculated values of the hydrogen-bonding component are plotted against the temperature and pressure, as shown in Figure 1. The components are much less affected by pressure, and while it is not apparent by the plot, the δ_h_ increases with increasing pressure. In the considered temperature and pressure range, the solvents are in the liquid phase, and similar temperature and pressure effects can be observed in Figure 2 in the work of Williams^27^ for liquid CO_2_.

### 3.2 Model Building

The difficulty that might arise in the description of GASF-based optical resolution with a single model is that the process is composed of three subprocesses (salt formation, precipitation, and extraction). It is straightforward that no general model can be established which gives a good description of the selectivity in any GASF processes independently of the applied solvent or racemate or resolving agent. However, if a model is found to be appropriate to describe the selectivity of a GASF process, it may be assumed that the parameters in that model are those that generally affect selectivity in different GASF processes. If it is known which parameters affect the process the most, the study of a yet uninvestigated system (with other racemates, other resolving agents, and other solvents) or optimization of an already investigated system becomes more energy- and material-efficient and simpler.

Data obtained in experiments of GASF is evaluated to find a relationship between the Hansen solubility parameters and selectivity of the process. Mathematical model building is performed, where the candidate parameters to be included in the model are temperature, pressure, and the Hansen solubility parameters. The idea is that the Hansen components may refer to the effect of the solvent mixture (i.e., solubility of the compounds) on the chemical equilibrium and the precipitation and the extraction phase, while the effects of the operating parameters (i.e., temperature and pressure) may be realizing mostly in connection with the chemical equilibrium. However, the Hansen components depend on the operating parameters due to the temperature and pressure dependency. Also, the Hansen components are correlated in the experiments due to the similarity of the formulas for temperature and pressure correction. The observed extent of the correlations depends highly on the experimental design, and a well-chosen design may decrease it considerably. The dependency of the predictors in regression analysis is called multicollinearity. The consequences of multicollinearity are increased values and increased uncertainty of the coefficient, which may even result in coefficients estimated with the wrong sign. Accordingly, when multicollinearity is present, the interpretation of the effects of the parameters based on the fitted model might be untrue. Despite this phenomenon, the overall goodness of fit of the description of the process is unaffected by the presence of multicollinearity. Although a model with the satisfying capability to describe a GASF process is already an important result, the intention in this paper is to advance even further and identify the true nature of the effects and to find a model which may be used to predict the selectivity. This may be achieved by decreasing the effects of multicollinearity. For this purpose, different statistical remedies may be used, for example, ridge regression and LASSO.^43^ These methods make the estimation of the regression line biased and by doing so, the coefficients estimated with a bias become less sensitive to the effects of multicollinearity. By eliminating potential effects of multicollinearity, the true nature of the effects (true sign and magnitude of the coefficients) may be revealed. To estimate the ridge model, R “glmnet” package^44^ was used.

#### 3.2.1 Experiments with Mandelic Acid

The four series of experiments from which the data are obtained are summarized in Table 1, while the data can be found in the Supporting Information tables. The data are evaluated in two groups. One group contains the experiments with mandelic acid, while the other one contains the experiments with ibuprofen as the racemate.

The data set of the experiment with mandelic acid is rather small, and the selectivity changes are in the narrow range (0.3–0.5). Thus, the information that can be obtained from this data set is limited. The evaluation of the mandelic acid experimental data aims to study the descriptive power of a potentially found simple model containing the Hansen components and to obtain a ridge regression that might reveal the true nature of the effects.

When the ordinary least-squares (OLS) method is used to estimate the regression line from the data obtained in the experiments with mandelic acid, the following model is obtained
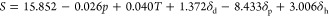
35where *S* is the selectivity, *p* is the pressure (MPa), *T* is the temperature (°C), and δ_d_, δ_p_, and δ_h_ are the Hansen components (MPa^0.5^). The effect of pressure is included in the model; however, it was found to be non-significant (*p* = 0.24). The estimates have high uncertainty and the relatively high coefficients of the Hansen parameters [meaningless to state that 1 unit change in δ_p_ changes selectivity (which is bounded between 0 and 1) by 8.5 unit] indicate severe nature of multicollinearity. The reason for that may be the relatively small number of experiments, the structure of the experimental design, and the lack of variability in the values of selectivity (0.3–0.5). The correlations of the predictors are given in Table 7. Variables with estimated correlation of greater than 0.3 or smaller than −0.3 may be considered to be correlated, while correlation of 0.92 (Table 7) indicates strong correlation.

**Table 7:** Correlation Coefficients of the Continuous Variables

	*T*	*p*	δ_d_	δ_p_	δ_h_
*T*	1.00	0.57	0.38	0.11	0.26
*p*		1.00	0.78	0.50	–0.61
δ_d_			1.00	0.92	–0.43
δ_p_				1.00	–0.32
δ_h_					1.00

When ridge regression is applied with the ridge parameter, λ of 0.0004 (the extent of bias introduced into the estimation of the regression line), which was chosen based on the ridge traces, the following model is obtained
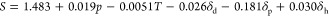
36

Besides the shrinkage of the coefficients toward zero, which is a consequence of ridge regression, the signs of the coefficients of temperature, pressure, and δ_d_ are changed, compared to the OLS model.

**Figure 3:**
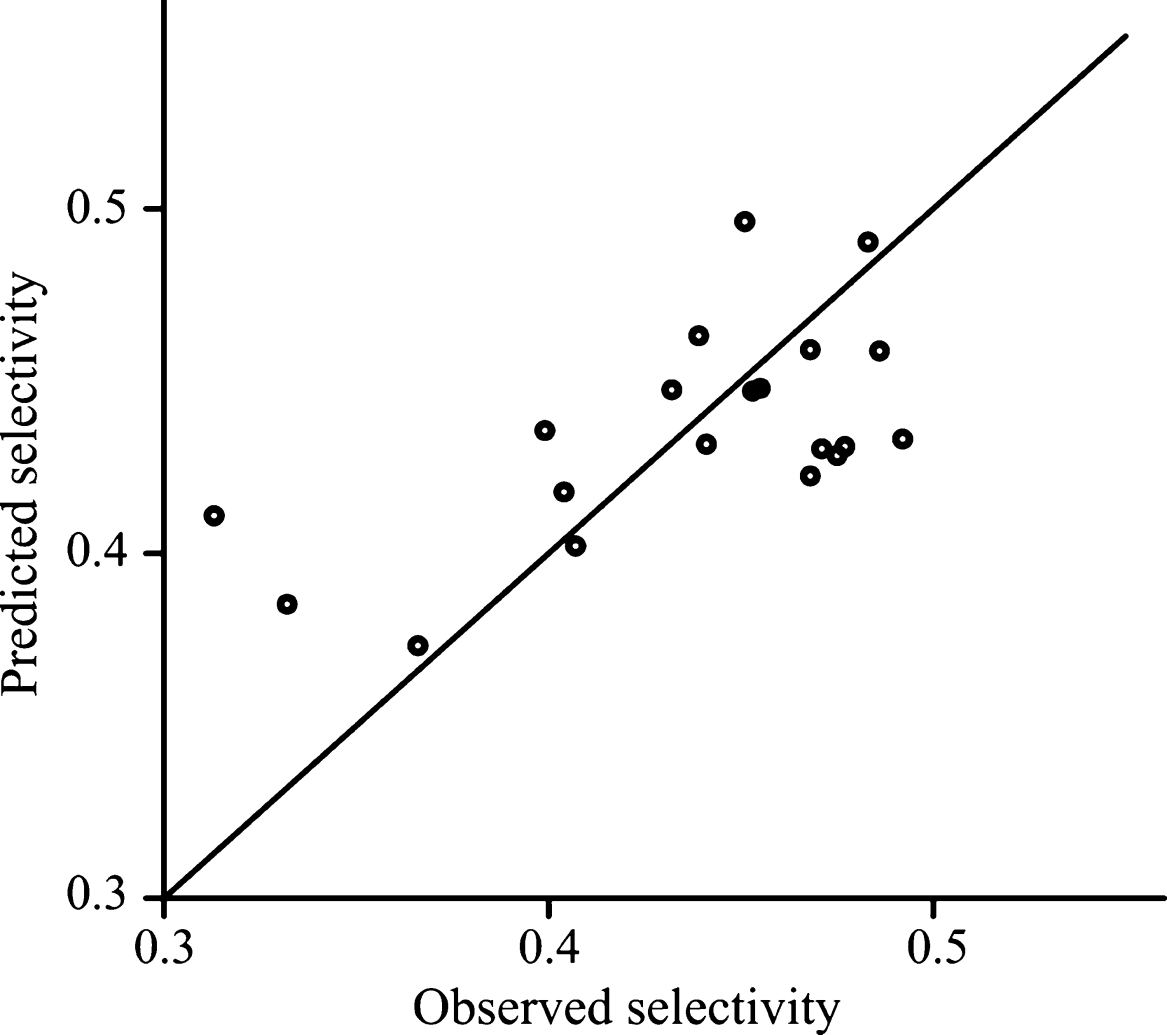
Goodness-of-fit using the ridge model for experiments with mandelic acid.

Figures 2 and 3 show the predicted selectivity against the observed values for each model. The bias that is introduced by ridge estimation distorts the satisfying correlation between the observed and predicted values found in the case of OLS model. However, the plot of the ridge model suggests that the introduced bias is not too great, and the deviations from the line representing the perfect correlation are still acceptable.

Although the results obtained from the OLS and ridge models are less reliable due to the nature of the data sets (especially because of the selectivity which changes in a narrow interval), it can be stated that a simple model that contains the effects of temperature, pressure, and the Hansen components is appropriate to describe the selectivity within the investigated design space. The ridge model might reveal the true nature of the effects (sign and magnitude); however, it cannot be stated with high confidence in this case due to the nature of the data set. The intention of the evaluation of this data set is to demonstrate that a simple model with good descriptive power can be constructed to describe the selectivity of a GASF process.

#### 3.2.2 Experiments with Ibuprofen

The data set of the experiments with ibuprofen is more appealing. The selectivity changes in a wide interval, and the number of experiments is large. Thus, by the statistical evaluation of the data set, more reliable results may be obtained, and the predictive power of the ridge model can be investigated as well.

For the experiments with ibuprofen, the following model was found to be appropriate to describe selectivity, using the OLS method
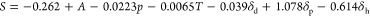
37where *S* is the selectivity, *p* is the pressure (MPa), *T* is the temperature (°C), δ_d_, δ_p_, and δ_h_ are the Hansen components (MPa^0.5^), and *A* is an alcohol-dependent parameter: *A* = 0 for methanol, *A* = 0.124 for ethanol, and *A* = 0.196 for propanol. The effect of δ_d_ is included in the model, but it was found to be non-significant (*p*-value = 0.47). Due to the high correlations present between the predictors (Table 8), the estimates of the true effects of the parameters in the description of selectivity are not necessarily reliable.

**Table 8:** Correlation Coefficients of the Continuous Variables

	*T*	*p*	δ_d_	δ_p_	δ_h_
*T*	1.00	–0.06	0.76	0.71	0.54
*p*		1.00	–0.56	–0.48	–0.50
δ_d_			1.00	0.94	0.83
δ_p_				1.00	0.87
δ_h_					1.00

**Figure 4:**
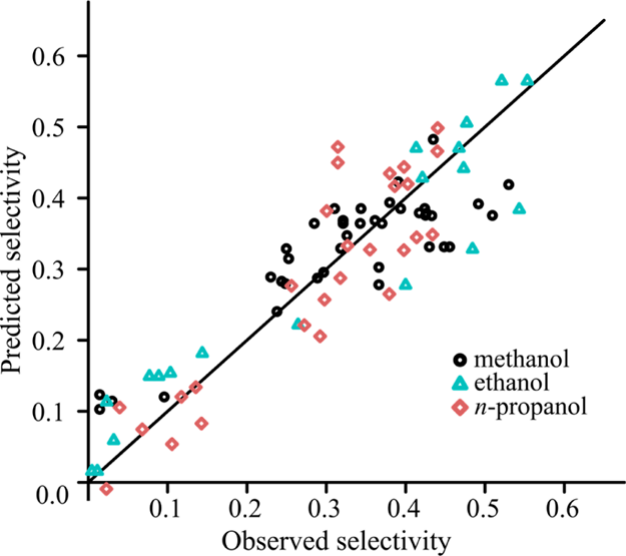
Goodness-of-fit using the OLS model for experiments with ibuprofen.

**Figure 5:**
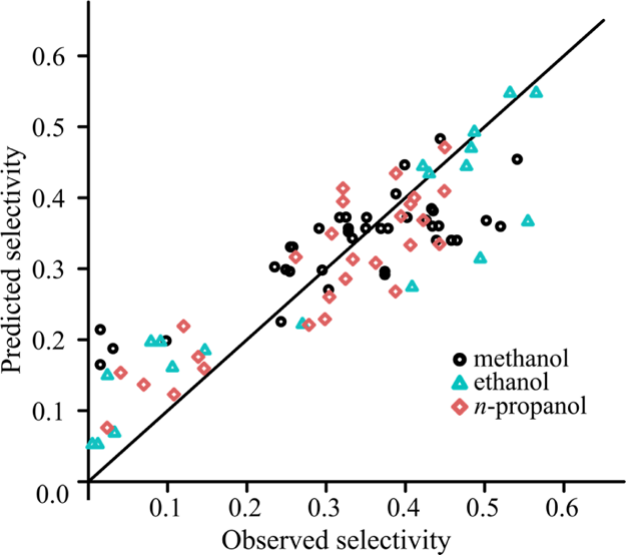
Goodness-of-fit using the ridge model for experiments with ibuprofen.

For example, because of the temperature and pressure correction of δ_p_, with decreasing temperature δ_p_ increases (eq 13) and thus it may be that the effect of δ_p_, which is found to be positive, is actually the effect of temperature being realized. Thus, it cannot be reliably stated that increment of δ_p_ is increasing selectivity independently of the temperature. To decrease the potential effects of multicollinearity and thus increase the reliability of the identification of the true nature of the effects, ridge regression was applied with λ of 0.005 obtained from the cross-validation method. The estimated ridge model is obtained as
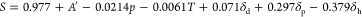
38where *A*′ = 0 for methanol and *A*′ = 0.0037 for ethanol, and *A*′ = −0.0045 for propanol. The model suggests that the selection of the alcohol has a neglectable effect on selectivity when methanol, ethanol, and *n*-propanol are considered. It should be noted that hypothesis tests on the ridge coefficients cannot be performed, that is, statistical significance cannot be investigated. It is worth noting that the sign of the coefficient of δ_d_ is changed when ridge regression is applied. The goodness-of-fit can be observed in Figures 4 and 5. The plots show the predicted selectivity against the observed values for the least-squares model and the ridge model. The plot of the ridge model looks less appropriate, and it is the effect of the bias that is introduced in the ridge estimation. The purpose of this plot is to show that the introduced bias is not too great, and the model remained meaningful.

It can be stated that the goodness-of-fit of the ridge model is acceptable with a maximum deviation of 0.2. It should be noted that the results for the different solvents were obtained in a wide range of time, and thus, uncontrolled factors may affect the variability to a great extent.

**Figure 6:**
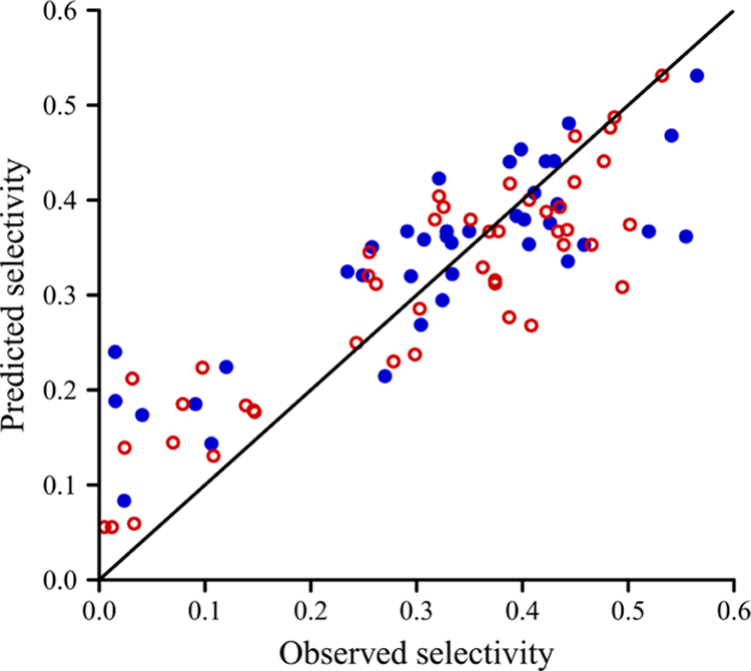
Selectivity predicted by the training set against the observed values of the training set (red circle) and the validation set (blue filled circle).

To examine the predictive accuracy of the ridge model, 40 observations were selected randomly as the validation set, and the remaining 46 points were used as the training set. The selectivity obtained from the ridge model with λ = 0.005 estimated from the training set was plotted against all of the observed values (Figure 6). It can be stated that the predictive accuracy for new observations is acceptable of the ridge model, with a maximum deviation of around 0.25 for the observed training and validate set.

#### 3.2.3 Discussion of the Experiments

It was found that a simple model is appropriate to describe selectivity in each of the investigated experiments. The descriptive power is satisfying (with a maximum deviation of around 0.15) which suggests that the most important parameters are in fact temperature, pressure, and the Hansen components. If the aim is to optimize, for example, the selectivity of the ibuprofen resolution, the found model may be applied. Using eqs 37 and 38, it can be stated reliably that the pressure and temperature are to be decreased to increase selectivity. When ridge regression is applied, the dependency of the effects in the estimated model decreases, that is, one can estimate the effects of the Hansen components independently (ideally) from the effect of pressure and temperature and the effects of the other Hansen components. That is, based on eq 38 it can be stated that regarding the Hansen components, the optimal selectivity can be approached by increasing δ_d_ and δ_p_ and decreasing δ_h_. For example, when δ_h_ is decreased by increasing the temperature (Figure 1), the change in the Hansen component and the change in the temperature both affect the selectivity presumably according to the coefficients in the ridge regression. In this experiment, it is inconvenient according to the ridge model to increase the Hansen components by increasing the pressure as pressure has a negative effect on selectivity. However, decreasing temperature (which increases selectivity) may be used to increase the Hansen components and thus increase selectivity. The most convenient way to change the values of the Hansen components may be the selection and the concentration of the co-solvent (or even the selection of the supercritical fluid). In the experiment with ibuprofen, a solvent with a larger δ_d_ and δ_p_ and smaller δ_h_, that is a solvent with less hydrogen-bonding (*n*-alcohol) characteristic, would presumably increase the selectivity. The effect of the co-solvents (independently of their effect on the Hansen components) is not obvious. Although it was found by the ridge regression that the difference between the solvents is negligible, it can be assumed that the solvent in fact affects the selectivity. The solvent molecules take part in the crystallization process, and they might be incorporated into the crystals as well. It might be the case that the difference of the molecular structure in methanol, ethanol, and *n*-propanol is not large enough to be detectable with satisfying certainty based on the data set at hand. When new co-solvents are applied, *A* (the effect of the co-solvent in the models) might change to a great extent. Nevertheless, when a process is investigated already, the models can be estimated, and optimization can be applied by changing the parameters in the model accordingly to its estimated effect.

The effects of the Hansen components differ in the two experiments, which is expected, as the solubility of the dissolved compounds, that is, the racemate and the formed salts, differ in the two experiments. The effects of the Hansen components are always based on the solubility of the dissolving compounds in the solvent mixture; therefore, there is not a general effect of the component that may be considered if the racemate or the resolving agent varies. However, it can be assumed that for any racemate and resolving agent, the most important parameters still will be the Hansen components. The same assumption can be made for the operating parameters: the found effects cannot be taken universally as they depend on the racemate and the resolving agent (supposedly due to the salt formation reaction mostly) as well. While the true nature of the effects of the Hansen components and operating parameters are unknown, the investigation of a yet-uninvestigated process may become more efficient as it is known that there are five parameters to be considered in the study of the process: temperature, pressure, and the Hansen components. With this information, one can apply designed experiments, considering the five parameters in the construction of the design, obtaining the most information possible from the performed experiments.

The models constructed in this paper may have limited applicability. This is because, in reality, it is not the Hansen components of the solvent that is of importance, but the difference between the Hansen parameters of the solvent and the solutes, that is, the Hansen “distance,” Ra. That is, while in the investigated region, the linear effect of the Hansen parameters of the solvent mixture is appropriate to describe the solubility-related part of GASF, at a wider region, the effects of the parameters may become non-linear. It depends on the solvent, the racemate, and the resolving agent as well when the limit of the applicability of the linear model is reached. Also, it should be noted that the case of GASF-based resolution is rather complicated as not only a single dissolved compound should be considered but a whole system, containing the unreacted enantiomers, resolving agents, and the formed salts as well. The fact, that despite these phenomena, there is still a place for simple models to describe the GASF process, gives hope in further application of the mathematical model building approach to help study and optimize GASF systems.

## 4 Conclusions

Mathematical model building with the purpose of description of selectivity of GASF-based optical resolution is performed in this paper. It was found that a simple model containing the operating parameters (temperature and pressure) and the Hansen solubility parameters is appropriate to describe selectivity with a satisfying maximum of 0.15 deviation between the measured and predicted values. Although the nature of the effects of the parameters are not universal, presumably these are the parameters that affect generally GASF processes. The usefulness of such a simple model is apparent in two areas: at the study of a yet-uninvestigated GASF system and at the optimization of an investigated GASF system. Based on the constructed models, investigation of new GASF processes can be achieved by designed experiments which is the most efficient method. The knowledge of the parameters affecting selectivity the most and the estimated model containing these parameters enables us to approach optimal selectivity more conveniently by changing the settings according to the coefficients in the model. For that, the correlations between the parameters are to be decreased which can be achieved with a well-chosen experimental design or application of ridge regression, which was applied in this paper. Application of the Hansen components requires correction of temperature and pressure. The formula in the literature for the hydrogen-bonding Hansen component is not substance specific. In this paper, a new formula is suggested for methanol, ethanol, and *n*-propanol which differentiates between the substances and thus provide a more accurate correction of temperature and pressure. The apparent difference, that can be observed between the temperature effects on the different alcohols, suggests that the generalization that is used in the formula of Williams^22^ is an inappropriate approximation.

## Supporting Information Available

Experiments with mandelic acid as the racemate and methanol as the co-solvent; experiments with ibuprofen as the racemate and methanol as the co-solvent; experiments with ibuprofen as the racemate and ethanol as the co-solvent; and experiments with ibuprofen as the racemate and *n*-propanol as the co-solvent (PDF)
