# Supplementary material for: New Formula for the Hydrogen-Bonding Hansen Component of Methanol, Ethanol, and n-Propanol for Non-ambient Conditions—Application in Gas Antisolvent Fractionation-Based Optical Resolution
Source: ACS Omega. 2021 Jul 12;6(29):18964–74. doi: 10.1021/acsomega.1c02223 (PMC8320073; doi:10.1021/acsomega.1c02223)
Supplement: Supplementary file 1 — ao1c02223_si_001.pdf [file ao1c02223_si_001.pdf]

New formula for the hydrogen-bonding Hansen component of methanol, ethanol and *n*-propanol for non-ambient conditions – application in gas antisolvent fractionation based optical resolution

Máté Mihalovits[a]*, Márton Kőrösi[a] and Edit Székely[a]

[a]Department of Chemical and Environmental Process Engineering, Budapest University of Technology and Economics, Budapest, Hungary

*Contact:
mihalovits.mate@vbk.bme.hu
Budapest University of Technology and Economics, Department of Chemical and Environmental Process Engineering
Hungary, 1111. Budapest, Műegyetem rakpart 3.

Supplementary

In the tables p is pressure, T is temperature, V_{CO2} is the molar volume of $scCO_2$ taken from NIST Chemistry Webbook, $V_{alcohol}$ is the molar volume of the alcohol calculated as described in *Section 2.2.3*, $\Theta_{alcohol}$ is the volume fraction of the alcohol in the mixture, $\delta_{d(mix)}$, $\delta_{p(mix)}$ and $\delta_{h(mix)}$ are the Hansen components for the mixture and S is the selectivity.

Table S1. Experiments with mandelic acid as racemate and methanol as co-solvent

p (MPa)	T (°C)	V_{CO2} (cm³/mol)	$V_{alcohol}$ (cm³/mol)	$\Theta_{alcohol}$	$\delta_{d(mix)}$ ($MPa^{0.5}$)	$\delta_{p(mix)}$ ($MPa^{0.5}$)	$\delta_{h(mix)}$ ($MPa^{0.5}$)	S
16	45	57.91	40.83	0.080	10.0506	5.7451	5.9332	0.4750
16	45	57.91	40.83	0.042	9.8443	5.4748	5.2795	0.4386
20	45	54.15	40.65	0.091	10.8824	5.9860	6.2673	0.4320
12	45	66.91	41.03	0.076	8.5542	5.3818	5.5688	0.4554
16	35	53.21	40.38	0.086	11.0854	6.0013	6.3199	0.4039
16	55	64.61	41.30	0.091	8.9759	5.5707	5.8098	0.4923
16	45	57.91	40.83	0.076	10.0315	5.7200	5.8724	0.4411
16	45	57.91	40.83	0.078	10.0393	5.7302	5.8972	0.4707
20	45	54.15	40.65	0.078	10.8218	5.8956	6.0454	0.4860
16	55	64.61	41.30	0.077	8.8849	5.4674	5.5708	0.4533
16	45	57.91	40.83	0.109	10.2072	5.9502	6.4292	0.4072
16	35	53.21	40.38	0.081	11.0605	5.9635	6.2253	0.4685
20	45	54.15	40.65	0.077	10.8198	5.8926	6.0380	0.4681

20	55	58.32	41.11	0.127	10.2159	6.0584	6.6114	0.3125
12	35	57.37	40.57	0.041	9.9556	5.4976	5.3698	0.3991
20	35	50.84	40.21	0.041	11.4845	5.8094	5.6478	0.4509
20	55	58.32	41.11	0.040	9.7513	5.4470	5.1656	0.4832
20	35	50.84	40.21	0.120	11.8017	6.3509	7.0153	0.4773
12	40	61.32	40.80	0.114	9.6491	5.8578	6.4502	0.3322
12	37	58.81	40.66	0.116	10.0946	5.9702	6.6027	0.3660

Table S2. Experiments with ibuprofen as racemate and methanol as co-solvent

p (MPa)	T (°C)	V_{CO2} (cm^3/mol)	$V_{alcohol}$ (cm^3/mol)	$\Theta_{alcohol}$	$\delta_{d(mix)}$ ($MPa^{0.5}$)	$\delta_{p(mix)}$ ($MPa^{0.5}$)	$\delta_{h(mix)}$ ($MPa^{0.5}$)	S
15	45	59.32	40.88	0.052	9.57	4.64	5.49	0.2911
21	45	53.00	40.61	0.053	10.91	4.89	5.77	0.3741
13	45	63.45	40.98	0.050	8.85	4.49	5.31	0.4017
13	45	63.45	40.98	0.050	8.85	4.49	5.32	0.4261
13	45	63.45	40.98	0.050	8.85	4.49	5.31	0.3172
13	45	63.45	40.98	0.050	8.85	4.49	5.31	0.3510
15	45	59.32	40.88	0.045	9.53	4.59	5.37	0.4331
15	45	59.32	40.88	0.045	9.54	4.59	5.38	0.4353
10	45	88.33	41.13	0.041	6.02	3.82	4.51	0.4390
11	45	72.97	41.08	0.048	7.53	4.21	4.99	0.5195
12	45	66.91	41.03	0.051	8.33	4.40	5.21	0.5016
13	45	63.45	40.98	0.052	8.86	4.51	5.36	0.4337
14	45	61.09	40.93	0.053	9.26	4.59	5.44	0.4423
15	45	59.32	40.88	0.052	9.57	4.64	5.49	0.3775
16	45	57.91	40.83	0.053	9.85	4.70	5.57	0.3330
17	45	56.75	40.79	0.054	10.08	4.74	5.62	0.2554
18	45	55.76	40.74	0.060	10.32	4.83	5.77	0.3742
19	45	54.91	40.70	0.048	10.45	4.77	5.60	0.2578
20	45	54.15	40.65	0.053	10.64	4.84	5.72	0.2946
21	45	53.00	40.61	0.052	10.91	4.88	5.75	0.2544
15	35	54.00	40.43	0.052	10.68	4.84	5.78	0.3986
15	55	67.35	41.36	0.049	8.25	4.36	5.09	0.3029
10	55	135.39	41.62	0.049	3.86	3.25	3.90	0.0313
10	35	61.74	40.66	0.051	9.14	4.55	5.45	0.4439
20	55	58.32	41.11	0.052	9.75	4.67	5.45	0.2431
20	35	50.84	40.21	0.053	11.47	4.98	5.95	0.3255
10	55	135.39	41.62	0.055	3.93	3.31	4.02	0.0153
10	55	135.39	41.62	0.045	3.82	3.22	3.84	0.0976
20	45	54.15	40.65	0.052	10.64	4.83	5.71	0.2490
15	45	59.32	40.88	0.052	9.57	4.64	5.49	0.3497
10	45	88.33	41.13	0.041	6.02	3.82	4.51	0.4583
10	55	135.39	41.62	0.040	3.77	3.17	3.75	0.0150
10	45	88.33	41.13	0.041	6.02	3.82	4.51	0.4654
15	45	59.32	40.88	0.052	9.58	4.65	5.50	0.3687
20	45	54.15	40.65	0.052	10.64	4.83	5.70	0.2346

15	45	59.32	40.88	0.052	9.58	4.65	5.50	0.3283
15	45	59.32	40.88	0.026	9.42	4.43	5.04	0.5409
15	45	59.32	40.88	0.039	9.50	4.54	5.27	0.3882
15	45	59.32	40.88	0.054	9.58	4.66	5.52	0.3283
15	45	59.32	40.88	0.052	9.57	4.64	5.49	0.3285

Table S3. Experiments with ibuprofen as racemate and ethanol as co-solvent

**p (MPa)**	**T (°C)**	**V_{CO2} (cm^3/mol)**	**$V_{alcohol}$ (cm^3/mol)**	**$\Theta_{alcohol}$**	**$\delta_{d(mix)}$ ($MPa^{0.5}$)**	**$\delta_{p(mix)}$ ($MPa^{0.5}$)**	**$\delta_{h(mix)}$ ($MPa^{0.5}$)**	**S**
**10**	45	88.33	59.23	0.122	6.86	4.11	5.62	0.0330
**10**	40	70.01	58.91	0.121	8.53	4.48	6.06	0.1060
**15**	40	56.41	58.61	0.121	10.60	4.87	6.50	0.0240
**20**	45	54.15	58.63	0.122	11.05	4.95	6.54	0.0120
**15**	35	54.00	58.31	0.121	11.10	4.96	6.62	0.0790
**20**	45	54.15	58.63	0.122	11.05	4.95	6.54	0.0050
**15**	35	54.00	58.31	0.121	11.10	4.96	6.62	0.0910
**10**	35	61.74	58.60	0.035	9.07	4.30	5.08	0.5650
**15**	45	59.32	58.92	0.031	9.47	4.36	5.03	0.4770
**10**	35	61.74	58.60	0.035	9.07	4.30	5.08	0.5320
**15**	45	59.32	58.92	0.031	9.47	4.36	5.03	0.4220
**15**	40	56.41	58.61	0.030	10.06	4.47	5.18	0.4870
**20**	35	50.84	58.03	0.030	11.39	4.69	5.46	0.4830
**20**	40	52.40	58.33	0.030	10.98	4.63	5.35	0.4300
**15**	45	59.32	58.92	0.052	9.61	4.46	5.34	0.5546
**15**	45	59.32	58.92	0.066	9.70	4.53	5.55	0.4944
**15**	45	59.32	58.92	0.077	9.77	4.57	5.70	0.4084
**15**	45	59.32	58.92	0.091	9.86	4.64	5.91	0.2701
**15**	45	59.32	58.92	0.102	9.93	4.69	6.06	0.1469

Table S4. Experiments with ibuprofen as racemate and *n*-propanol as co-solvent

**p (MPa)**	**T (°C)**	**V_{CO2} (cm^3/mol)**	**$V_{alcohol}$ (cm^3/mol)**	**$\Theta_{alcohol}$**	**$\delta_{d(mix)}$ ($MPa^{0.5}$)**	**$\delta_{p(mix)}$ ($MPa^{0.5}$)**	**$\delta_{h(mix)}$ ($MPa^{0.5}$)**	**S**
**15**	45	59.32	75.41	0.045	9.56	4.34	5.14	0.3212
**15**	35	54.00	74.70	0.059	10.75	4.57	5.60	0.3880
**15**	40	56.41	75.05	0.057	10.21	4.47	5.43	0.4112
**20**	35	50.84	74.37	0.058	11.52	4.69	5.73	0.4226
**20**	40	52.40	74.71	0.057	11.11	4.62	5.61	0.4062
**10**	40	70.01	75.41	0.075	8.15	4.10	5.21	0.4430
**10**	45	88.33	75.79	0.080	6.43	3.72	4.81	0.2782
**15**	35	54.00	74.70	0.118	11.07	4.70	6.33	0.1201
**10**	35	61.74	75.05	0.102	9.53	4.41	5.84	0.2616
**10**	35	61.74	75.05	0.057	9.22	4.29	5.28	0.4497
**20**	45	54.15	75.06	0.054	10.68	4.55	5.46	0.3625

**20**	45	54.15	75.06	0.098	10.91	4.65	5.99	0.0407
**20**	40	52.40	74.71	0.107	11.36	4.74	6.21	0.1459
**15**	45	59.32	75.41	0.092	9.86	4.45	5.72	0.2984
**15**	45	59.32	75.41	0.109	9.97	4.50	5.92	0.1386
**15**	45	59.32	75.41	0.123	10.06	4.53	6.10	0.1080
**15**	45	59.32	75.41	0.137	10.14	4.57	6.27	0.0236
**15**	45	59.32	75.41	0.084	9.81	4.44	5.62	0.3041
**15**	45	59.32	75.41	0.068	9.71	4.40	5.43	0.3337
**15**	45	59.32	75.41	0.051	9.60	4.35	5.21	0.3944
**15**	45	59.32	75.41	0.040	9.53	4.32	5.07	0.3212
**15**	45	59.32	75.41	0.076	9.76	4.41	5.52	0.3244
**15**	45	59.32	75.41	0.082	9.80	4.43	5.59	0.3875
**15**	45	59.32	75.41	0.058	9.65	4.37	5.30	0.3070
**15**	45	59.32	75.41	0.040	9.53	4.32	5.08	0.4492
**15**	45	59.32	75.41	0.119	10.03	4.52	6.05	0.0698
**15**	45	59.32	75.41	0.045	9.56	4.34	5.15	0.4064
